# Behavioral and Neuropathological Phenotyping of the Tau58/2 and Tau58/4 Transgenic Mouse Models for FTDP-17

**DOI:** 10.3390/life13102088

**Published:** 2023-10-20

**Authors:** Debby Van Dam, Femke Valkenburg, Kristof Van Kolen, Isabel Pintelon, Jean-Pierre Timmermans, Peter Paul De Deyn

**Affiliations:** 1 Laboratory of Neurochemistry and Behavior, Experimental Neurobiology Unit, University of Antwerp, Wilrijk, 2610 Antwerp, Belgium; femke.valkenburg@uantwerpen.be; 2 Department of Neurology and Alzheimer Center Groningen, University Medical Center Groningen, University of Groningen, 9713 GZ Groningen, The Netherlands; 3 Neuroscience Department, Janssen Research and Development, 2340 Beerse, Belgium; kvkolen@its.jnj.com; 4 Laboratory of Cell Biology and Histology, Department of Veterinary Sciences, University of Antwerp, 2610 Antwerp, Belgium; isabel.pintelon@uantwerpen.be (I.P.); jean-pierre.timmermans@uantwerpen.be (J.-P.T.)

**Keywords:** tauopathy, frontotemporal dementia, Alzheimer’s disease, motor dysfunction, cognitive deficits, behavioral disinhibition, Morris water maze

## Abstract

**Simple Summary:**

The tau protein normally functions to maintain the stability of microtubules, the cellular cytoskeleton that provides structure and shape to eukaryotic cells. Tau protein malfunction is a core feature of a group of neurodegenerative diseases labeled ‘tauopathies’ that include, among others, Alzheimer’s disease and frontotemporal dementia, with parkinsonism linked to chromosome 17 (FTDP-17). In these disorders, abnormally hyperphosphorylated tau is accumulated in intraneuronal tangles, disturbing normal cellular function and acting as the basis of neurofibrillary degeneration and dementia. FTDP-17 is indeed a form of frontotemporal dementia or frontotemporal degeneration characterized by a loss of nerve cells in areas of the brain called the frontal and temporal lobes. Over time, this cell loss affects personality, behavior, language, and movement. Valid animal models are vital to enhance our understanding of molecular disease mechanisms and as preclinical tools to identify novel treatment targets and to screen the efficacy of novel drug candidates or other therapeutic approaches. We report the validity of two genetically modified mouse models that express a mutated form of human tau protein associated with FTDP-17. With aging, both lines developed progressively worsening tau-related pathology, cognitive decline, and behavioral changes reminiscent of the symptom profile of FTDP-17 patients.

**Abstract:**

Background: The Tau58/2 and Tau58/4 mouse lines expressing 0N4R tau with a P301S mutation mimic aspects of frontotemporal dementia and parkinsonism linked to chromosome 17 (FTDP-17). In a side-by-side comparison, we report the age-dependent development of cognitive, motor, and behavioral deficits in comparison with the spatial-temporal evolution of cellular tau pathology in both models. Methods: We applied the SHIRPA primary screen and specific neuromotor, behavioral, and cognitive paradigms. The spatiotemporal development of tau pathology was investigated immunohistochemically. Levels of sarkosyl-insoluble paired helical filaments were determined via a MesoScale Discovery biomarker assay. Results: Neuromotor impairments developed from age 3 months in both models. On electron microscopy, spinal cord neurofibrillary pathology was visible in mice aged 3 months; however, AT8 immunoreactivity was not yet observed in Tau58/4 mice. Behavioral abnormalities and memory deficits occurred at a later stage (>9 months) when tau pathology was fully disseminated throughout the brain. Spatiotemporally, tau pathology spread from the spinal cord via the midbrain to the frontal cortex, while the hippocampus was relatively spared, thus explaining the late onset of cognitive deficits. Conclusions: Our findings indicate the face and construct validity of both Tau58 models, which may provide new, valuable insights into the pathologic effects of tau species in vivo and may consequently facilitate the development of new therapeutic targets to delay or halt neurodegenerative processes occurring in tauopathies.

## 1. Introduction

Neurodegenerative tauopathies are age-related diseases that include most notably Alzheimer’s disease (AD), frontotemporal dementia with parkinsonism linked to chromosome 17 (FTDP-17), corticobasal degeneration (CBD), and progressive supranuclear palsy (PSP). All these diseases share the common feature of the accumulation of aggregates of hyperphosphorylated microtubule-associated protein tau (MAPT), which results in neurofibrillary tangles (NFTs), one of the hallmarks of AD, the most prominent tauopathy [1,2]. Since the burden of NFTs and their distribution through the brain correlates strongly with the stage of cognitive deficits in AD [3] and other tauopathies [4,5], it is clear that tau is of importance in the onset and progression of neurodegenerative dementias. Furthermore, mutations in the tau gene can cause inherited forms of FTDP-17 [6,7], and currently, over 60 pathogenic mutations in the MAPT gene have been associated with various autosomal dominantly inherited tauopathies [8]. However, the underlying mechanism and the way in which the accumulation of tau can cause neurodegeneration are not yet clear. Also, FTDP-17, CBD, and PSP are characterized by motor deficits that also present in Parkinson’s disease, including bradykinesia, tremors, and rigidity, which occur in the early stages of the disease [9].

To investigate the mechanism by which tau exerts its effects, various transgenic tau mouse models that are based on mutations linked to human tauopathies have been generated [2,7,10,11,12,13,14,15]. The observed phenotype in tau mouse models depends on the tau mutation itself, the promoter(s) used, and the composition of the inserted tau isoform(s) [16,17]. The P301S mutation, located in exon 10 of the MAPT gene, causes early-onset FTDP-17 with rapidly progressing and often severe clinical signs [18,19,20,21,22,23]. To investigate the pathomechanisms of P301S mutations, transgenic P301S mice have been generated, among which are the Tau58/2 and Tau58/4 lines that are subject of this study. The Tau58 lines were originally developed by Novartis Institutes for Biomedical Sciences (Basel, Switzerland), and aspects of face, construct, and predictive validity have been appraised in other studies [24,25,26,27,28,29,30,31,32,33,34,35,36,37]. However, none of these studies focused on the age-dependent development and progression of cognitive, behavioral, and motor deficits in relation to the spatial-temporal neuropathological alterations in both P301S mouse models. Murine models that overexpress human-mutated tau genes are essential tools to screen drug candidates that aim to intervene in the formation and deposition of insoluble tau species promoted by increased tau phosphorylation. The side-by-side comparison of both P301S models enables us, in a unique manner, to investigate the effects of the location of the insertion in the mouse genome and possible different resulting phenotypes, which will allow researchers to make a substantiated decision about which model and disease stage to choose for future research projects.

## 2. Materials and Methods

### 2.1. Tau58 Mouse Lines

The generation of the Tau58/2 and Tau58/4 mouse lines was previously described [27,38]. In brief, the 0N4R human tau gene containing a P301S mutation under the control of a mThy1.2 promoter was expressed in mice with a C57BL/6J background. The size of the inserted minigene (TAU0N4R P301S) was 1149 bp, existing of 383 amino acids with a weight of 40 kDa. No additional polyadenylation sites were inserted. Male heterozygous mice were weaned four weeks after birth and housed with wild-type (WT) littermates in mixed genotype groups. Food and water were supplied ad libitum. Custom primers (forward primer: TAA AGA GAG GGG CTG AGG TAT T; reverse primer: GTG GCG ATC TTC GTT TTA CCA, Biolegio, Nijmegen, The Netherlands) were used for genotyping by PCR analysis. The size of the expected band was 442 kb.

Experimenters were blinded to the genetic status of the animals during testing. For all behavioral tests, male heterozygous (HET) mice of the Tau58/2 and Tau58/4 models aged 3, 6, 9, and 12 months were used in addition to WT littermates of both models of the corresponding age as controls. Due to increased motor disturbances and morbidity with aging, mice aged 15 and 18 months could not be evaluated in behavioral tests. For immunohistochemistry (IHC) and electron microscopy (EM), mice aged 3, 6, 9, 12, 15, and 18 months were used (Table A1). For sarkosyl extractions and the MesoScale Discovery (MSD) assay of tau, HET and WT mice aged 17–20 months were used. All motor tests were performed during the light (inactive) phase of the animals, while exploration and cognitive assessments were performed during the dark (active) phase of the animals. All mice were acclimatized to the experimental room at least one hour before conducting the experiments. Experiments were carried out in compliance with the European Community Council Directive on the protection of animals used for scientific purposes (2010/63/EU) and were approved by the Animal Ethics Committee of the University of Antwerp.

### 2.2. Progression of Tau Pathology

#### 2.2.1. Immunohistochemistry and DAB Semi-Quantification

Coronal sections of the spinal cord, hippocampus, and frontal cortex (5 µm, paraffin-embedded) of Tau58/2 and 58/4 mice aged 3, 6, 8, 9, and 12 months (and 18 months for histology and electron microscopy) were dissected and subsequently prepared for immunohistochemical staining. After deparaffinization in xylene and ethanol, sections were pre-incubated in 1% H_2_O_2_ for 30 min. For AT270 staining, antigen retrieval with a 0.01 M citrate buffer (pH = 6) was performed before pre-incubation. Sections were washed in tris-buffered saline (TBS) and blocked for 30 min with 4% normal swine serum in TBS and 1% bovine serum albumin (BSA). After blocking, sections were incubated overnight with a primary antibody (AT8, 1:10,000, produced in-house; AT270, 1:10,000, ThermoFisher Scientific, Waltham, MA, USA, MN1050; and HT7, 1:5000, ThermoFisher Scientific, MN1000) at 4 °C in TBS and 1% BSA. Sections were washed again with TBS and incubated with biotinylated anti-mouse IgG (1:200, Amersham, UK, RPN1001) or polyclonal rabbit anti-mouse IgG (1:500, ThermoFisher Scientific, 31188) in TBS and 1% BSA at room temperature for 30 min. Finally, all sections were washed with TBS and incubated with the avidin-biotin-peroxidase complex (Vectastain ABC kit, Vector Laboratories, Newark, CA, USA, PK-6100) for 30 min and visualized with 3,3′-Diaminobenzidine (DAB, Sigma, D-5637).

Semi-quantification of DAB-stained immunoreactivity to HT7, AT8, and AT270 in the frontal cortex, hippocampus, and midbrain of Tau58/2 and Tau58/4 mice was performed with QuPath bioimage analysis software (version 0.4.4), allowing for the quantification of DAB-positive pixels versus the total number of pixels per investigated area (Figure A2) [39].

#### 2.2.2. Electron Microscopy of Tau Pathology

Conventional electron microscopy was performed on ultrathin slices of cortical samples fixed overnight with 4% glutaraldehyde. Postfixation was carried out in 2% osmium tetroxide, and blocks were subsequently embedded in araldite. Slices were double stained with uranyl acetate and lead citrate and examined with an FEI Philips CM10 transmission electron microscope (FEI, Eindhoven, The Netherlands) at 60 kV.

For immunoelectron microscopy (immuno-EM), cortical blocks of 1 mm^3^ were fixed for 2 h in 2% paraformaldehyde with 0.05% glutaraldehyde and embedded in Unicryl (EMS, Hatfield, PA, USA). Subsequently, ultrathin sections were cut (Ultracut UC7, Leica, Solms, Germany) and collected on pioloform-coated nickel grids. Rinsing was performed by using a BSA-c buffer consisting of 0.01 M PBS, pH 7.4, supplemented with 0.1% BSA-c (Aurion, Wageningen, The Netherlands). Blocking of the residual aldehyde groups was performed with 50 mM glycine in 0.01 M PBS (pH 7.4) and a blocking solution with normal goat serum to inhibit non-specific interactions (Aurion). Incubation of the grids with primary anti-phospho-tau antibody AT8 (produced in-house), diluted in a BSA-c buffer, occurred overnight at 4 °C. Subsequently, sections were incubated for 2 h with a secondary antibody, anti-mouse IgGs coupled with 10-nm gold, diluted in a BSA-c buffer. The sections were then rinsed in a BSA-c buffer, 0.01 M PBS, and ultrapure H_2_O. Postfixation of the grids was carried out using 2% glutaraldehyde in 0.01 M PBS followed by a short contrasting step with 2% uranyl acetate. Negative control samples were omitted for these experiments. Grids were examined using a Tecnai G2 Spirit BioTWIN TEM (FEI, Eindhoven, The Netherlands).

#### 2.2.3. Sarkosyl Extractions and MesoScale Discovery Assay of Tau

Sarkosyl extractions and the MesoScale Discovery (MSD) assay were performed as previously described [40]. In brief, mouse brain tissue of 17- to 20-month-old Tau58/2 mice (*n* = 13), Tau58/4 mice (*n* = 17), and WT littermates (*n* = 7) was weighed and homogenized in 6 volumes of homogenization buffer containing 10 mM Tris, 800 mM NaCl, 1 mM EGTA, and 10% sucrose (pH 7.4). The homogenate was centrifuged at 27,000× *g* (20 min), and 1% N-lauroylsarcosine was added to the supernatant. After 90 min, solutions were centrifuged at 184,000× *g* (1 h), and the pellet containing the sarkosyl-insoluble material was resuspended in a homogenization buffer.

For the MSD assay, coating antibody (AT8) was diluted in PBS (1 µg/mL), aliquoted into MSD plates (30 µL per well), and incubated overnight at 4 °C (L15XA, MSD, Rockville, MD, USA). After washing (5 × 200 µL of PBS with 0.5%Tween-20), the plates were blocked with 0.1% casein in PBS and washed again as before. Samples and standards were diluted in 0.1% casein in PBS, and plates were incubated overnight at 4 °C. After washing, the SULFOTAG™-conjugated detection antibody (AT8) in 0.1% casein in PBS was added and incubated for 2 h at room temperature while shaking at 600 rpm. After the final washing procedure, 150 µL of 2× buffer T (MSD) was added, and plates were read with an MSD imager. Raw signals were normalized against a standard curve consisting of 16 dilutions of a sarkosyl-insoluble preparation from a postmortem AD brain (ePHF) and were expressed as arbitrary units (AU) ePHF. An ‘in-house’ developed application for automated data analysis was used.

### 2.3. SHIRPA Primary Screening

The SHIRPA primary screening is a comprehensive behavioral and functional test battery designed to enable fast screening of mouse phenotypes, as most of the measures in the battery are susceptible to change after gene modifications or physiological alterations. The procedure enables identification of neuromuscular abnormalities encompassing global disturbances in gait, posture, and muscle tone as well as abnormalities in motor control and coordination [41]. The screening furthermore includes measurements of weight and general body condition. The SHIRPA primary screening was conducted as previously described [42].

### 2.4. Assessment of Neuromotor Function

Motor function evaluation was based on gait analysis, the wire suspension test, stationary beam test, and the accelerating rotarod as previously described [43,44].

#### 2.4.1. Gait Analysis

Gait characteristics (stride length, toe span, and track width) were analyzed by applying ink to the hind paws of the animals. Mice were then allowed to walk on a strip of paper in a brightly lit walk lane (width: 4.5 cm; length: 40 cm) towards a dark goal box. At least two complete gait patterns from each mouse were obtained.

#### 2.4.2. Wire Suspension Test

During the wire suspension test of grip strength and endurance, the front paws of the mouse were positioned on a horizontal steel wire (diameter: 0.6 mm) suspended at a height of 46 cm above the workbench. Test parameters were the latency to the first fall and the number of falls during the 2-min assessment period.

#### 2.4.3. Stationary Beam Test

The stationary beam test of equilibrium and balance was performed on a wooden beam (diameter: 25 mm; length: 110 cm) covered with a layer of masking tape to provide a firm grip. The beam was divided into 11 segments and placed at a height of 38 cm above a cushioned bench. The ends of the beam were shielded with cardboard to prevent the mice from escaping. Testing commenced by placing an animal in the middle of the beam. The number of segments crossed (four-paw criterion), the latencies before falling, and the number of falls were measured for four trials with a cutoff period of 1 min per trial and an intertrial interval of 10 min.

#### 2.4.4. Accelerating Rotarod

Equilibrium, balance, and motor coordination were tested on an accelerating rotarod apparatus (Panlab, Barcelona, Spain). After two acclimatization trials with a maximum duration of 2 min each at a constant speed (4 rpm), the mouse was placed on the rotating rod for four test trials, during which the rotation speed gradually increased from 4 to 40 rpm (inter-trial interval: 1 min). The time an animal stayed on the rod (latency to fall from the rod) was measured during the test trials with a cutoff period of 5 min per trial.

### 2.5. Assessment of Cognitive Function

#### 2.5.1. Passive Avoidance Learning

Passive avoidance learning was assessed using a compartmentalized step-through box during the dark (active) phase of the light/dark cycle [45]. The setup consisted of a brightly lit compartment connected to a dark compartment via a sliding door. The mice were placed in the brightly illuminated compartment, and the sliding door connecting the compartments was opened after a period of 5 s. Upon complete entry into the dark compartment (four-paw criterion), the mice received a mild foot shock (0.3 mA for 1 s). Exactly 24 h later, the latency to re-enter the dark compartment was timed up to 300 s, and the percentage of animals that did not reach this criterion was compared between experimental groups.

#### 2.5.2. Morris Water Maze Learning and Memory

Hippocampus-dependent visuospatial learning and memory were assessed by means of a hidden-platform Morris water maze (MWM) test [45,46]. The setup consisted of a circular pool (diameter: 150 cm; height: 30 cm) filled with opaque water with a temperature of 25 °C. A round, plastic platform was placed in one quadrant of the MWM, submerged below the surface. The acquisition phase consisted of eight blocks of four trials per day starting from four different positions in a semi-random order and a 15-min inter-trial interval. If mice were unable to reach the platform within 120 s, they were placed on the platform where they stayed for 15 s before being returned to their home cages. The acquisition phase was followed 4 days later by a probe trial in which the platform was removed from the MWM, and the animals were allowed to swim freely for 100 s. During acquisition training and the probe trials, the animals’ trajectories were recorded (EthoVision, Noldus, Wageningen, The Netherlands). During the training trials, the escape latency (duration) to the platform and path lengths (distance) were measured. During the probe trial, performance was measured as the percentage of time spent in each quadrant of the MWM and the number of times the mice crossed over the position where the platform was previously located.

### 2.6. Assessment of Activity, Exploration, and Anxiety-like Behavior

The applied tests included the evaluation of spontaneous, horizontal locomotion over a 47 h period in a home cage, an open field test to assess exploration in a novel environment, and the elevated plus maze to assess anxiety-like behavior as previously described [45,47,48].

#### 2.6.1. 47 h Cage Activity Recordings

Cage ambulatory activity patterns were assessed for individually housed animals using standard mouse cages (length: 22.5 cm; width: 16.7 cm; height: 14 cm). Cages were positioned between three infrared sensors (two oriented perpendicular and one parallel to the cage) positioned 3 cm above bedding material to detect horizontal movement. Cages were placed in an enclosure equipped with ventilation to maintain an optimum ambient temperature and illumination to simulate the light/dark cycle of the mice. The number of beam interruptions was subsequently recorded over a 47-h period in 30-min bins, taking into account that the initial hours that the mouse is placed in this new environment are to be considered as exploration. Recordings started at 16.00 h on day 1 and ended at 15.00 h on day 3.

#### 2.6.2. Open Field Test

Open field behavior was recorded in a brightly lit arena (length: 50 cm; width: 50 cm). Mice started from the same corner of the arena and were allowed 1 min of adaptation before the 10 min recording period was initiated. A computerized video-tracking system (Ethovision) was used to record trajectories and calculate path length and number of entries in the center circle (diameter: 25 cm) or the corners (7 cm × 7 cm) of the arena.

#### 2.6.3. Elevated Plus Maze

The elevated plus maze (EPM) is a cross-shaped maze with two open arms and two closed arms (length: 30 cm; width: 5 cm, and a height of 15 cm in the closed arms) and a central area (5 cm × 5 cm). At the start of the 5 min trial (EthoVision), mice were placed in the central area facing an open arm in the direction away from the experimenter. The measured parameters were total path length and velocity in the entire EPM, frequency, time spent, path length, velocity, and immobility (settings: lower immobility threshold 5%; upper mobile threshold 95%) in the open and closed parts of the EPM.

### 2.7. Statistical Analysis

Statistical analyses were performed in SPSS 26 (IBM, Armonk, NY, USA) and GraphPad Prism 6.0 (GraphPad Software Inc, La Jolla, CA, USA) with a probability level set at 95%. The mean scores during the trials in the MWM test and latencies in the accelerating rotarod test were investigated by means of a two-way repeated measures analysis of variance (RM-ANOVA) with a Bonferroni post hoc test. To compare the means of paired parameters, the two-tailed Student’s *t*-test was applied. For MSD data, an ANOVA with Bonferroni post hoc test was performed. The data were considered statistically significant if *p* < 0.05.

## 3. Results

### 3.1. Phosphorylated Tau Load Increases with Aging in Tau58/2 and Ta58/4 Mice

**Figure 1:**
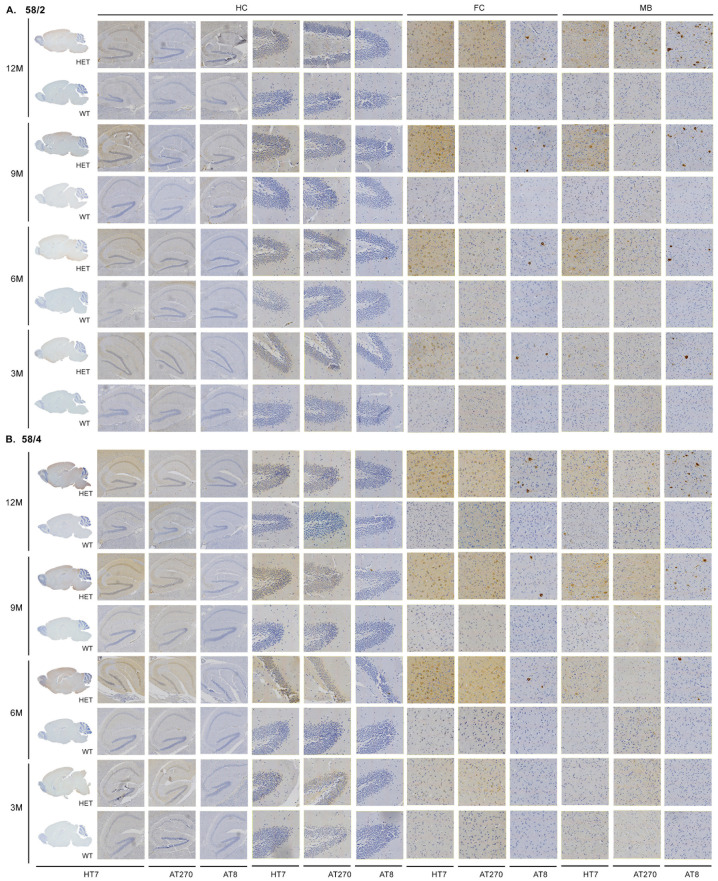
Immunohistochemical (IHC) analysis on the different age groups shows a clear spatiotemporal pattern. (**A**) IHC performed on the Tau58/2 mouse model. HT7 (whole brain sagittal, hippocampus (HC), frontal cortex (FC), and midbrain (MB)), AT8, and AT270 (hippocampus, frontal cortex, midbrain; magnification 40×) staining indicate a spatiotemporal spread from the spinal cord via the midbrain to the frontal cortex and hippocampus. (**B**) IHC performed on the Tau58/4 mouse model. HET = heterozygous, WT = wild-type.

**Figure 2:**
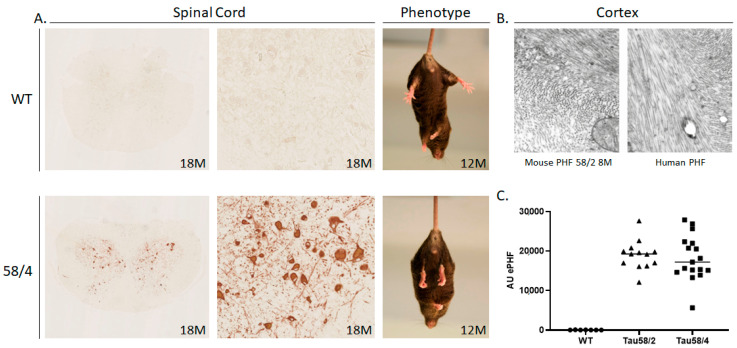
Inclusions of phosphorylated tau and intraneuronal tau fibrils in the spinal cord and cortex. (**A**) Left panels: coronal sections of the spinal cord of 18-month-old Tau58/4 and WT mice stained with the AT8 antibody. Upper and lower left panel magnification: 2.5×, center panels magnification: 20×. Right panels: the resulting phenotype is characterized by the presence of hindlimb clasping, which is observed from the age of 9 months and older in the Tau58/4 model [27]. (**B**) Comparison of mouse neuropathology in the Tau58/2 model (left panel) with human pathology (right panel) in cortical sections. NFTs appear strikingly similar in both sections (magnification in both sections: 52,500×). (**C**) Brain homogenates of aged mice were analyzed with an AT8/AT8 aggregation selective MSD assay, indicating a significantly higher level of tau aggregation in both models versus WT mice. HET = heterozygous, WT = wild-type, NFT = neurofibrillary tangles, PHF = paired helical filaments, AU = arbitrary units.

**Figure A1:**
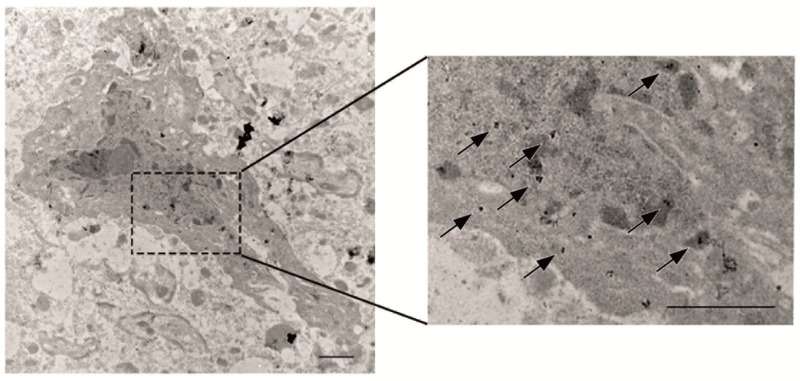
AT8 immuno-EM staining for phosphorylated tau (phosphorylation sites: serine 202 and threonine 205) with 10-nm gold particles in a pyramidal neuron located in the frontal cortex of a 12-month-old Tau58/2 mouse. Inset: arrows denote the intraneuronal gold particles, indicative of the presence of phosphorylated tau. Scale bar indicates 1 µm.

**Figure A2:**
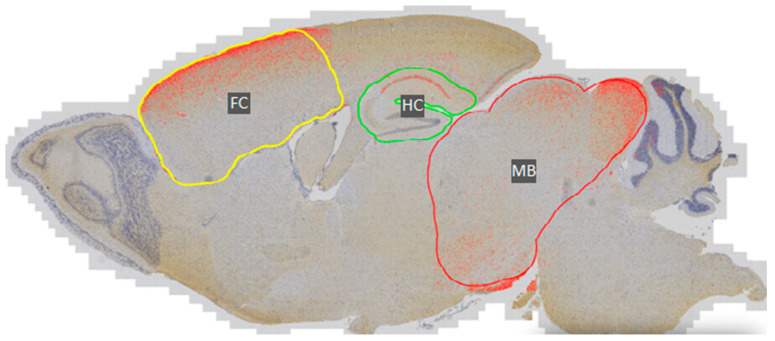
Delineation of the brain regions selected for semi-quantification of immunoreactivity. Magnification: 40×. FC = frontal cortex, HC = hippocampus, MB = midbrain.

**Figure A3:**
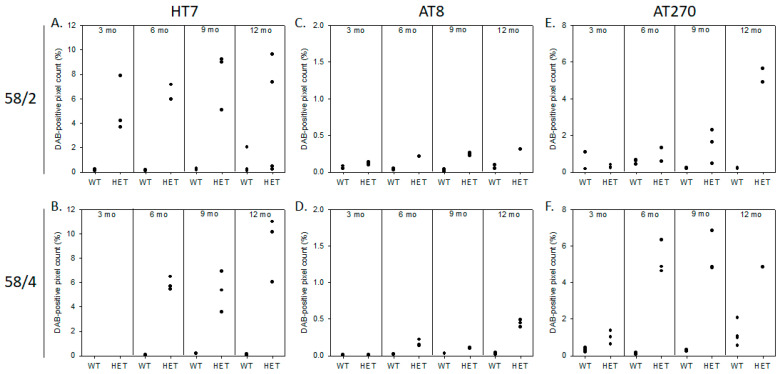
Semi-quantification of tau immunoreactivity in frontal cortex. The number of DAB-positive pixels versus the total number of pixels based on HT7, AT8, and AT270 immunostaining in the frontal cortex of Tau58/2 and Tau58/4 mice was determined using QuPath. (**A**) HT7 staining in WT and 58/2 mice. (**B**) HT7 staining in WT and 58/4 mice. (**C**) AT8 staining in WT and 58/2 mice. (**D**) AT8 staining in WT and 58/4 mice. (**E**) AT270 staining in WT and 58/2 mice. (**F**) AT270 staining in WT and 58/4 mice. DAB = 3,3′-Diaminobenzidine, HET = heterozygous, WT = wild-type.

**Figure A4:**
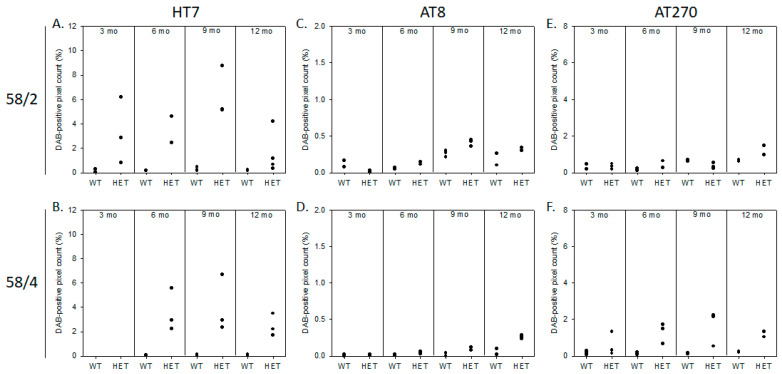
Semi-quantification of tau immunoreactivity in hippocampus. The number of DAB-positive pixels versus the total number of pixels based on HT7, AT8, and AT270 immunostaining in the frontal cortex of Tau58/2 and Tau58/4 mice was determined using QuPath. (**A**) HT7 staining in WT and 58/2 mice. (**B**) HT7 staining in WT and 58/4 mice. (**C**) AT8 staining in WT and 58/2 mice. (**D**) AT8 staining in WT and 58/4 mice. (**E**) AT270 staining in WT and 58/2 mice. (**F**) AT270 staining in WT and 58/4 mice. DAB = 3,3′-Diaminobenzidine, HET = heterozygous, WT = wild-type.

**Figure A5:**
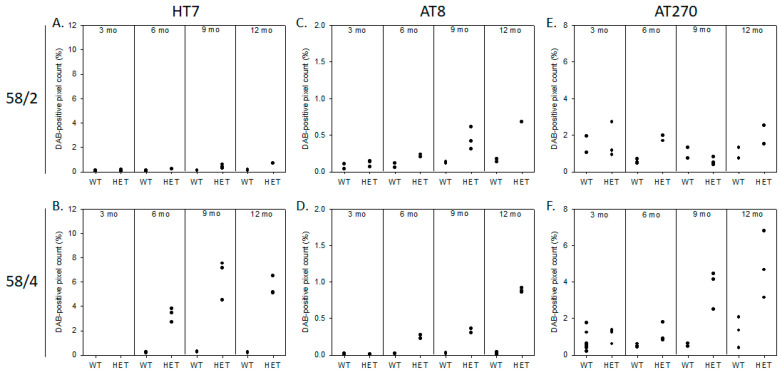
Semi-quantification of tau immunoreactivity in midbrain. The number of DAB-positive pixels versus the total number of pixels based on HT7, AT8, and AT270 immunostaining in the frontal cortex of Tau58/2 and Tau58/4 mice was determined using QuPath. (**A**) HT7 staining in WT and 58/2 mice. (**B**) HT7 staining in WT and 58/4 mice. (**C**) AT8 staining in WT and 58/2 mice. (**D**) AT8 staining in WT and 58/4 mice. (**E**) AT270 staining in WT and 58/2 mice. (**F**) AT270 staining in WT and 58/4 mice. DAB = 3,3′-Diaminobenzidine, HET = heterozygous, WT = wild-type.

**Figure A6:**
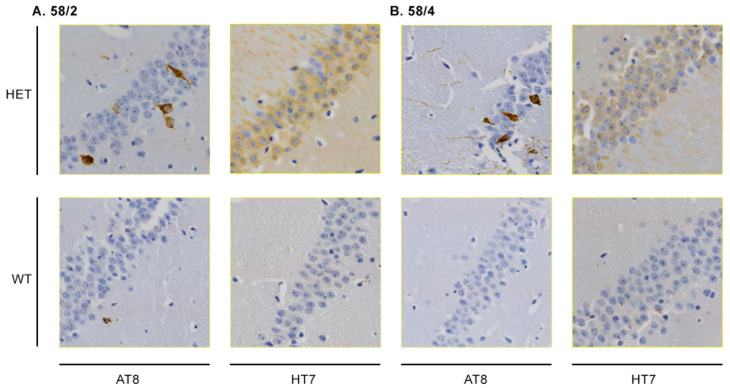
Detail of hippocampal tau pathology. At 12 months of age, tau pathology is visible most prominently via AT8 and HT7 staining in both 58/2 and 58/4 mice. (**A**) AT8 and HT7 staining in 58/2 mice. (**B**) AT8 and HT7 staining in 58/4 mice. Magnification: 40×. HET = heterozygous, WT = wild-type.

HT7 immunoreactivity for total human tau was first observed in the spinal cord of young Tau58/2 mice aged 3 months; occurrence in the 58/4 model started at the age of 6 months. Hyperphosphorylated tau, by means of AT8-positive staining, was first observed in the spinal cord and the frontal cortex of Tau58/2 and 58/4 mice aged 3 and 6 months, respectively (Figure 1 and Figure 2). From 9 months of age onward, tau pathology was visible in the midbrain in the Tau58/2 model (Table A2). From the age of 12 months, hyperphosphorylated tau was observed clearly in the frontal cortex as indicated by positive AT8 immunostaining and visible intraneuronally on (immuno-)EM (Figure 1, Figure 2 and Figure A1). From 12 months of age, AT8 and AT270 immunoreactivity were clearly visible not only in the frontal cortex but also in the midbrain and spinal cord, indicating a temporal pattern of hyperphosphorylation at the different epitopes of MAPT. Semiquantification of HT7-, AT8-, and AT270-based tau immunoreactivity in the frontal cortex, hippocampus, and midbrain is presented in Figure A3, Figure A4 and Figure A5, respectively. In addition, Figure A6 specifically presents tau pathology in the hippocampus at age 12 months. Total tau (HT7) and phosphorylated tau at epitopes Ser202/Thr205 (AT8) and at epitope Thr181 (AT270) showed a clear progression of immunostaining in HET mice of both lines with aging.

Sarkosyl extractions of total brain tissue combined with the MSD assay indicated significantly and analogously increased levels of aggregated hyperphosphorylated tau in the sarkosyl-insoluble fraction in both ages (17–20 months) of Tau58 models compared to WT mice (Figure 2C).

### 3.2. SHIRPA Primary Screening Indicates Age- and Genotype-Related Deficits in Both the Tau58/2 and 58/4 Models

**Figure 3:**
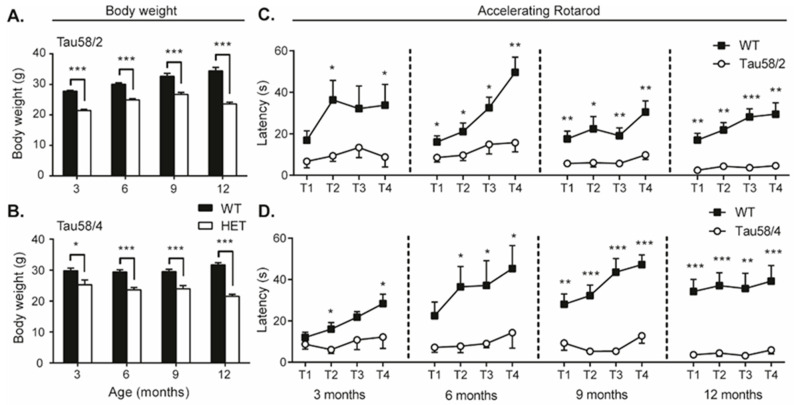
Evolution of body weight and neuromotor performance as assessed with the accelerating rotarod. (**A**) A significantly lower body weight was observed in HET animals in comparison with WT, age-matched littermates. In the Tau58/2 model, HET animals of 3 months (t(18) = 11.377, *p* < 0.001), 6 months (t(34) = 8.222, *p* < 0.001), 9 months (t(23) = 4.937, *p* < 0.001), and 12 months (t(20) = 9.114, *p* < 0.001) had a significantly lower body weight than WT animals. Furthermore, while an age-related increase in body weight was visible in WT animals, a decrease was observed in HET animals. (**B**) For the Tau58/4 model, all HET age groups had a significant lower weight than their corresponding WT controls (3 months (t(13.771) = 2.654, *p* = 0.019), 6 months (t(20) = 5.271, *p* < 0.001), 9 months (t(21) = 4.112, *p* < 0.001), and 12 months (t(29) = 10.728, *p* < 0.001)). (**C**,**D**) WT animals of both models scored significantly better during both the training phase (2 trials, T0a and T0b with a constant speed of 4 rpm and a cutoff time of 120s; not indicated in this figure) and the test phase (4 trials, T1, T2, T3, and T4, with a 5 min acceleration to 40 rpm). Furthermore, HET animals either fell from the apparatus or jumped, while WT animals kept on running. Two-way RM-ANOVA with Bonferroni post hoc comparison indicated that for all age groups of both mouse models, WT animals scored significantly better than HET animals (genotype interaction). The significance of each individual time point as indicated in the figure was determined with a two-tailed Student’s *t*-test. Data are presented as mean ± SEM. The used sample size is indicated in Table 1. * = *p* < 0.05, ** = *p* < 0.01, *** = *p* < 0.001. HET = heterozygous, WT = wild-type, RM ANOVA = repeated measures analysis of variance.

The SHIRPA primary screen revealed significant differences in the body weight of HET tau animals of both models compared to age-matched WT littermates (Figure 3A,B). Most prominent was the difference in weight in the oldest age group of 12 months (Tau58/2: t(20) = 9.114, *p* < 0.001; Tau58/4: t(29) = 10.728, *p* < 0.001). Furthermore, HET animals of both models performed inferiorly during motor tasks in the SHIRPA screen, indicative of motor impairment. In the 58/2 model, prominent significant differences versus controls were observed in the contact-righting reflex and the negative geotaxis from the age of 6 months on (contact-righting reflex: 6 months χ(3) = 9.257, *p* = 0.026; 9 months (3) = 16.987, *p* = 0.001; 12 months χ(3) = 10.895, *p* = 0.012; negative geotaxis: 6 months χ(3) = 16.444, *p* = 0.001; 9 months χ(1) = 15.385, *p* < 0.001; 12 months χ(3) = 18.543, *p* < 0.001). Tremors were observed in all age groups of Tau58/4 mice (3 months: χ(1) = 9.975, *p* = 0.001; 6 months: χ(1) = 10.267, *p* = 0.001; 9 months: χ(1) = 4.439, *p* = 0.035; 12 months: χ(2) = 13.839, *p* = 0.001), whilst this was not the case in Tau58/2 mice. Furthermore, a decrease in grip strength was found in 58/4 HET animals of all ages (3 months: χ(2) = 7.574, *p* = 0.023; 6 months: χ(3) = 16, *p* = 0.001; 9 months: χ(2) = 7.463, *p* = 0.024; 12 months: χ(2) = 17.168, *p* < 0.001). In both models, reduced locomotor activity was observed in 12-month-old HET animals (58/2: t(20) = 3.155, *p* = 0.005; 58/4: t(17) = 6.584, *p* < 0.001). Consequently, motor functioning was further investigated with the rotarod, wire suspension, and stationary beam tests as well as through gait pattern analysis. Phenotypically, HET animals aged >9 months presented with a hunched posture, moderate to severe scoliosis, and ungroomed fur. The SHIRPA screen did not indicate differences in the skin color of the paws of the mice, nor were there any differences in body temperature measured.

### 3.3. Tau58/2 and 58/4 HET Mice Exhibit Neuromotor Impairment Compared with Age- and Genotype-Matched Control Animals

The wire suspension test did not reveal significant differences in the number of falls between HET and controls of both tau models. These findings are possibly explained by the loss of body weight in the HET animals (Figure 3A,B), thus requiring less strength during the wire suspension test. However, in both the stationary beam test and the gait analysis, HET animals displayed age-dependent motor impairment compared with their WT counterparts (Table 1). In the stationary beam test, HET animals covered a lower number of segments (58/2: 6 months t(34) = 3.360, *p* = 0.002; 9 months, borderline significant; 12 months t(20) = 3.929, *p* = 0.001; 58/4: 3 months t(9) = 4.236, *p* = 0.002; 6 months t(20) = 3.403, *p* = 0.003; 9 months t(15) = 2.916, *p* = 0.010; 12 months t(16) = 2.916, *p* = 0.010), thus traveling a shorter distance, and had a higher number of falls than WT animals (Table 1). These differences were most prominent in the older age groups and were more pronounced in the Tau58/4 model than in the 58/2 model.

Gait analysis indicated that the stride length in HET animals was shorter in both models than in the WT control animals (Table 1). A decrease in stride length was observed bilaterally, with a significantly shorter length visible from 3 months in the Tau58/2 model (t(18) = 3.622, *p* = 0.002) and from 6 months in the Tau58/4 model (t(20) = 0.349, *p* < 0.001). Again, in accordance with the results found in the stationary beam test, an age-related increase in comparison with WT control animals was observed.

Neuromotor performance and equilibrium were also analyzed with the accelerating rotarod (Figure 3C). In all age groups, significant differences between HET and WT animals were observed in the fourth (i.e., final) trial (T4) and in the respective learning curves for all four trials (Tau58/2: 3 months F(1) = 21.878, *p* < 0.001; 6 months F(1) = 14.095, *p* = 0.001; 9 months F(1) = 14.541, *p* = 0.001; 12 months F(1) = 74.685, *p* < 0.001; Tau58/4: 3 months F(1) = 5.226, *p* = 0.035; 6 months F(1) = 10.958, *p* = 0.003; 9 months F(1) = 63.247, *p* < 0.001; 12 months F(1) = 71.672, *p* < 0.001). However, the results were confounded by the fact that HET animals did not always fall but instead jumped off the rod. Thus, the data obtained with the accelerating rotarod are biased by potential behavioral changes, such as stress and agitation, in tau HET mice.

### 3.4. Overexpression of Tau in HET Animals of the Tau58/2 and 58/4 Model Leads to a Decline in Cognitive Functioning

Passive avoidance testing did not reveal significant differences between HET and WT control littermates in either of the models in the latency to enter a previously visited compartment where a foot shock was delivered (Table 2).

**Figure 4:**
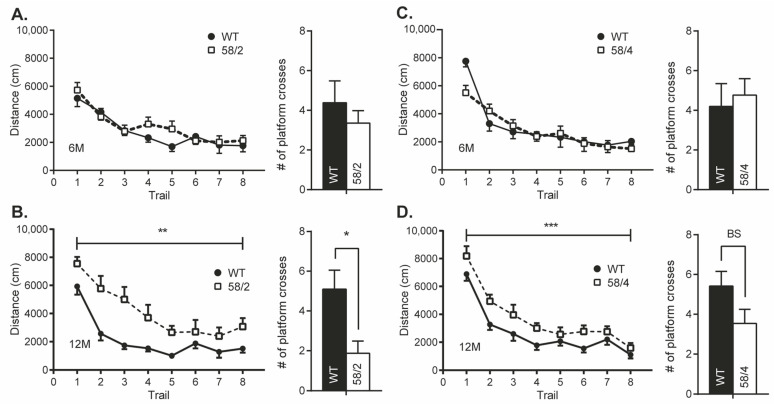
Performance of the Tau58/2 and 58/4 lines in the Morris water maze test for hippocampus-dependent visuo-spatial learning and memory. (**A**) Left panel: path length (distance) of Tau58/2 heterozygous (HET) mice (black dots) and wild-type (WT) control littermates (white squares) of 6 months of age during the acquisition trials in the MWM experiment. No significant differences were found in mice aged 6 months. Right panel: number of platform crossings during the probe trial of the MWM. (**B**) Left panel: path length of Tau58/2 HET mice and WT controls of 12 months of age during the acquisition trials in the MWM experiment. Two-way RM ANOVA indicated a significant difference between the learning curves of both genotypes (F(1) = 12.813; *p* = 0.002). Right panel: number of platform crossings during the probe trial of the MWM. Two-tailed Student’s *t*-test indicated that WT mice crossed the location of the removed hidden platform significantly more than HET mice aged 12 months. (**C**) Left panel: path length of Tau58/4 HET mice and WT control littermates aged 6 months during the acquisition trials of the MWM. Post hoc two-tailed Student’s *t*-test indicated a significant difference in the first trial as indicated by asterisks. Right panel: number of platform crossings during the probe trial of the MWM. WT and HET mice did not differ significantly in the number of platform crossings. (**D**) Left panel: path length of Tau58/4 HET mice and WT controls aged 12 months. Two-way repeated-measures ANOVA indicated a significant difference between the learning curves of both genotypes (F(1) = 15.471; *p* = 0.001). Right panel: number of platform crossings during the probe trial of the MWM. Two-tailed Student’s *t*-test indicated that WT mice crossed the location of the removed hidden platform more than HET mice aged 12 months, borderline significant (BS; *p* = 0.08). Each data point represents the summed mean (± SEM) across all performed trials or trial blocks. Significance is indicated by asterisks: * = *p* < 0.05, ** = *p* < 0.01, *** = *p* < 0.001. HET = heterozygous, WT = wild-type, MWM = Morris water maze, BS = borderline significant, RM ANOVA = repeated measures analysis of variance.

Significant genotype-based differences between 12-month-old mice of both Tau58 lines were observed in the MWM. HET mice of both models exhibited a slightly delayed learning curve compared to WT littermates (Tau58/2: F(1) = 12.813; *p* = 0.002; Tau58/4: F(1)= 15.471; *p* = 0.001), indicating that HET mice had deficits in visuospatial learning (Figure 4). At earlier ages, differences were not unambiguous for all parameters of the MWM test. Furthermore, the number of platform crossings during the probe trial was significantly lower in HET mice of the Tau58/2 model at the age of 12 months compared with WT littermates (t(15) = 2.822, *p* = 0.013). For the 58/4 model, differences were borderline significant (*p* = 0.08). Since age-dependent motor impairments would bias the outcome of cognitive tests requiring intact motor function, we were unable to investigate behavioral alterations in cohorts of male mice aged 15 and 18 months.

### 3.5. HET Animals of Both Tau58 Models Are More Active and Show Less Anxiety but Do Not Have an Altered 47 h Activity Profile

**Figure 5:**
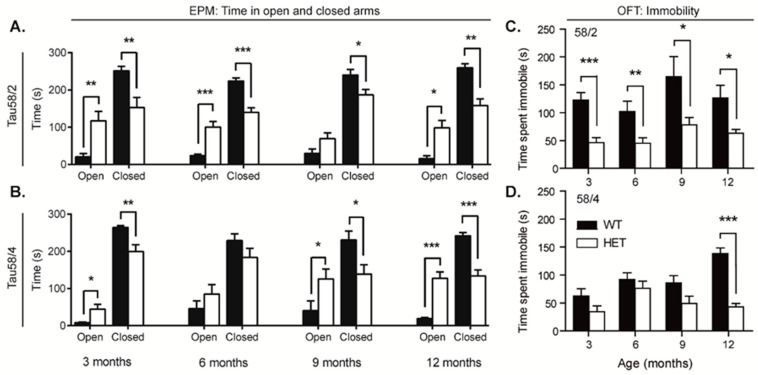
HET mice of both models spent more time in the open arms of the EPM and exhibited less immobility in the OFT. (**A**,**B**) Time spent in the open and closed arms of the elevated plus maze. HET animals of both models aged 12 months (58/2: t(13) = −3.278, *p* = 0.006; 58/4: t(25) = −2.293, *p* = 0.034) spent significantly more time in the open arms and less time in the closed arms (58/2: t(13) = 4.189, *p* = 0.001; 58/4: t(29) = 2.070, *p* = 0.047), indicating more exploratory and less anxiolytic behavior than WT littermates. These results are in agreement with results from the open field test, which indicated that HET animals of the Tau 58/4 model aged 9 and 12 months spent a significant longer time in the center circle of the open field (9 months: t(21) = −2.131, *p* = 0.045; 12 months: t(19) = −4.281, *p* < 0.001) and significantly less time in the corners (9 months: t(21) = 2.465, *p* = 0.022; 12 months: t(29) = 2.552, *p* = 0.016) in comparison with WT littermates. (**C**,**D**) Time spent immobile in the open field area during the open field test. WT animals of both models aged 12 months spent a significant longer percentage of time in an immobile state (freezing) compared with HET animals. For the 58/2 model, the difference at 12 months was smaller than that for the 58/4 model (t(29) = 3.928, *p* < 0.001). The immobility observed during the elevated plus maze indicated no significant differences. Data are presented as mean ± SEM. Statistical significance was determined with the two-tailed Student’s *t*-test. * = *p* < 0.05, ** = *p* < 0.01, *** = *p* < 0.001. HET = heterozygous, WT = wild-type, OFT = open field test, EPM = elevated plus maze.

To investigate alterations in behavior, the open field test and the elevated plus maze were conducted. In nearly all age groups of both models, HET animals tended to spend a significant longer time in the open arms of the elevated plus maze in comparison with WT controls (Table 2; Figure 5A,B). Moreover, the WT animals aged 12 months spent a significantly longer time in the closed arms of the maze (58/2: t(20) = 3.705, *p* = 0.001; 58/4 t(29) = 5.826, *p* = 0.001), indicating a higher degree of exploration and/or a lower degree of anxiety in the HET animals. In 12-month-old animals of the 58/4 model, the total path length was significantly higher in WT animals (t(29) = 3.152, *p* = 0.004), while the time spent in the open arms was still significantly higher in the HET animals (t(16.222) = −6.222, *p* < 0.001). Immobility during the elevated plus maze did not significantly differ between both genotypes in any of the age groups.

HET animals of both models spent a longer time in the center circle of the open field in comparison with WT littermates; this difference increased with age and was significant for all age groups above 3 months in the 58/4 model. Furthermore, HET animals of all age groups of both the 58/2 and 58/4 models spent significantly less time in an immobile state compared with WT controls (Figure 5C,D). While this difference in immobility was not observed in the elevated plus maze, a significant difference in the total covered distance was found for the 58/4 model at 12 months (t(29) = −3.106, *p* = 0.004) but not for the 58/2 model.

**Figure A7:**
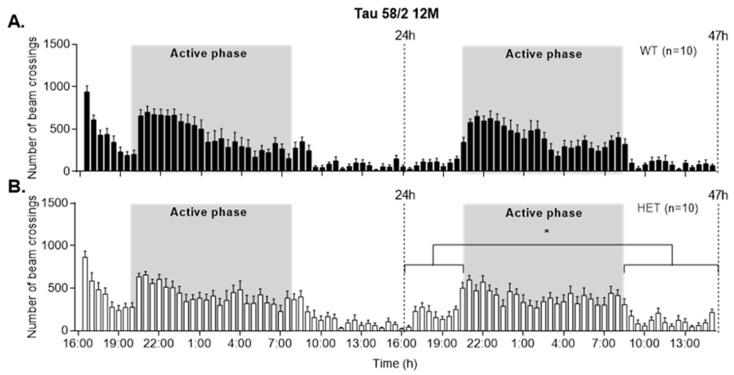
47 h cage activity recordings in the Tau58/2 mouse line. (**A**) Activity profile of WT mice (black bars) aged 12 months. (**B**) Recordings of HET mice (white bars) aged 12 months. Activity profiles of HET and WT mice look similar, though the difference in the active (i.e., dark) and inactive phase in HET mice looks less pronounced. Total number of counts did not differ between both genotypes. Activity during the active (light) phase of day two (16:00–20:00 h and 08:00–15:00 h) is significantly higher in HET animals compared with WT controls (two-tailed Student’s *t*-test, t18 = −2.334, *p* = 0.031). Each data point represents mean (± SEM) across all measured time points (30 min bins). HET = heterozygous, WT = wild-type. * indicates significance between the inactive and active phase (*p* < 0.05).

**Figure A8:**
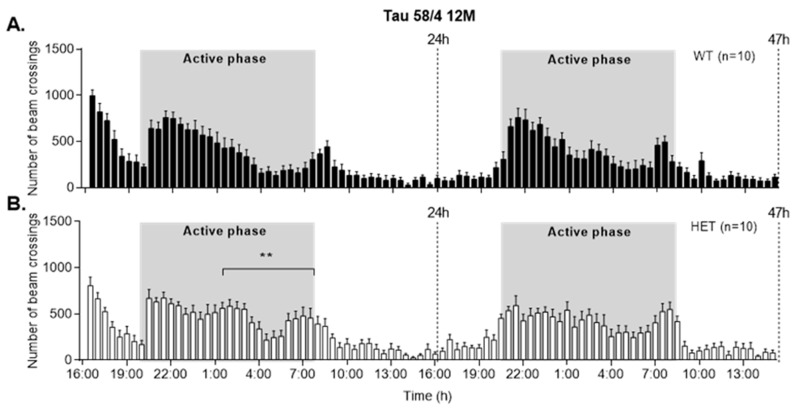
47 h cage activity recordings in the Tau58/4 mouse line. (**A**) Activity profile of WT mice (black bars) aged 12 months. (**B**) Recordings of HET mice (white bars) aged 12 months. HET mice are more active during the second half of the first night versus WT mice (two-tailed Student’s *t*-test, t18 = −3.060, *p* = 0.007, as indicated by **). This (hyper)activity corresponds with the increased distance observed in the open field test (Table 2; Figure 5).

Cage activity recordings indicated slight, albeit not statistically significant, differences in overall horizontal spontaneous locomotor activity for mice aged up to 9 months. At the age of 12 months, however, activity during the active (light) phase of day two was significantly higher in Tau58/2 HET animals compared with WT controls (t(18) = −2.334, *p* = 0.031) (Figure A7). For the Tau58/4 model, HET mice aged 12 months were more active during the second half of the first night (t(18) = −3.060, *p* = 0.007) (Figure A8).

## 4. Discussion

In this study, we performed a side-by-side comparison of two related P301S tauopathy mouse models. Other aspects of face validity as well as elements of construct and predictive validity have been studied before by our team and others [24,25,26,27,28,29,30,31,32,33,34,35,36,37]. None of the earlier studies, however, focused on the age-dependent development and progression of cognitive, behavioral, and motor deficits in relation to the spatial-temporal neuropathological alterations in both P301S mouse tauopathy models.

Both Tau58 lines develop tau-related pathology in a comparable spatiotemporal pattern, albeit with Tau58/2 mice being affected earlier in life compared to the Tau58/4 line. Aging Tau58 mice presented phenotypically with a hunched posture, scoliosis, mild eye irritation, and periocular loss of fur. Since eye irritation only occurred in HET animals at older ages (>6 months), we argue that this was not due to expression of the Pde6brd1 retinal degeneration allele, as reported by Lewis et al. [17], but most likely due to excessive self-grooming, analogous to the abnormal repetitive, obsessive compulsive disorder-like or self-harming behaviors [49] that are commonly observed in (bv)FTD patients [50,51].

Approximately 15% of FTDP-17 patients present motor symptoms [52], and significant tau-related pathology develops in the spinal cord, which prompted us to assess motor function in our animals by applying various setups in addition to the motor-related parameters included in the SHIRPA primary screen and the follow-up of clasping behavior. SHIRPA screening indicated progressive motor dysfunction, but not all neuromotor parameters were affected analogously in both mouse lines. Alterations in various SHIRPA neuromotor-related parameters were previously reported in the Tau58/2 line but with slightly different parameters significantly differing from WT mice in comparison to our SHIRPA results in the TAU58/2 line [26]. SHIRPA primary screen is a fully observational test and may therefore lack sensitivity, discriminative power, and reproducibility between facilities, hence explaining the differences between studies, but it is nevertheless an excellent initial phenotype screen that highlights phenotypic features requiring further specific analysis [53].

Deficits in rotarod performance have been described at ages as early as 2–3 months in the Tau58/2 model [24,26]. In a related P301S model, rotarod deficits were detected from the age of 5 months onward and progressed to a severe paraparesis at 6 months in homozygous animals [54]. Our experiments revealed significant differences in rotarod performance starting from the earliest investigated age of 3 months, with a further deterioration with aging in both models. Progression to paraparesis was not observed, with the advantage that in these models, behavioral experiments could still be conducted at various ages, in which a better understanding of the spatiotemporal spreading of tau pathology and its effects on motor function, cognition, and behavioral parameters could be obtained. However, the observed differences in the rotarod test cannot solely be attributed to motor function since HET mice of both models fell, as well as jumped, off the rod, thus implying an interfering behavioral (agitation) factor. Moreover, Tau58 mice displayed hindlimb clasping starting at 6 months in the 58/2 model and between 6 to 9 months in the 58/4 model. Hindlimb clasping is a feature that has been described in various other tauopathy mouse models with spinal cord tau pathology [17,54,55,56,57] and is considered a common manifestation of neurological or neurodegenerative diseases in mice [55]. Both Tau58 models displayed prominent clasping behavior from the age of 9 months, suggesting reduced muscle strength in the hindlimbs. The effect of this phenomenon on motor function was further investigated with gait analysis. Both Tau58/2 and 58/4 mice displayed an age- and genotype-dependent shorter stride in comparison with their WT littermates starting at the age of 3 months, a finding that is in accordance with results previously described in the Tau58/2 model [24]. Interestingly, no significant differences were found for the wire suspension test, in which forepaw muscle strength and endurance is tested [38]. We hypothesize that this is a consequence of the significantly lower body weight of the HET animals in comparison with the WT animals, as indicated by the SHIRPA screen. Therefore, the HET animals required less strength to keep hanging than the heavier control animals. Additionally, the observed muscle loss in the forepaws was visually clearly inferior to the muscle loss in the hindlimbs. An age-dependent decrease in body weight is frequently reported in various tauopathy mouse models [14], including previously in the Tau58/2 line [24,26], and might—at least partially—be related to the observed decrease in hindlimb muscle diameter in the Tau58/4 model [27], which was also observed in a related tauopathy mouse model expressing the P301L mutation in tau (4R0N) [58], but also, wasting and metabolic alterations may underlie the weight loss. Differences in body weight have also been described in human patients but mostly present as an increase in body weight due to binge eating [59,60]. Eating abnormalities have been observed in 60% of patients with bvFTD and include a wide range of disorders: increased appetite, hyperphagia or binge eating, lack of satiety, profound changes in food preferences, pica behavior, as well as gluttonous behaviors, for example, indiscriminate eating, stealing, and cramming food [59,60]. These eating disorders have been linked to atrophy in fronto/striatal areas and insula as well as in the hypothalamus [59,61]. Ingestive behavior has been limitedly studied in tauopathy mouse models. While lower food intake was reported in a particular P301L mouse model [62], increased food intake concomitant with a rise in resting metabolic rate was observed in another P301L mutation mouse model (Tg4510 mice) at age 7 months, while wasting was present in older animals with a considerable decrease in resting metabolic rate and food intake [63,64]. Interestingly, the Tg4510 model differs from other tau transgenic mice in the fact that significant tau pathology is present in the forebrain in comparison to the spinal cord [63,64]. Investigating ingestive behavior and metabolic state in relation to the spatiotemporal progression of tau pathology could also be of interest in the Tau58 lines.

To explore locomotion and equilibrium, we also applied the stationary beam paradigm, which has been described as being more sensitive than the rotarod [53], as indeed in the rotarod test mice are forced to move, while in the stationary beam test, movement is completely voluntary, which might make equilibrium problems more discernible. Significant differences were found at all ages for the Tau58/4 model but only in the older age groups for the Tau58/2 model, although borderline significant differences were noted in the youngest age groups. Applying a similar traversing beam challenge test, progressive worsening of performance was previously reported in Tau58/2 mice, albeit only two age groups (3 months vs. 10 months) were included [24].

As early symptoms of FTD typically involve behavioral alterations, changes in personality, and impairments in social interactions [51,65], various aspects of behavior were evaluated in both Tau58 models. We observed disinhibition in the EPM, where HET animals of both models displayed an altered exploration pattern and reduced anxiety in a novel environment in comparison with WT littermates, as indicated by a preference for the open arms versus closed arms. Tau58 mice of both lines spent more time in open arms and less time in closed arms, with virtually no difference in total exploration levels based on total distance traveled. The EPM paradigm is based on a fear and curiosity balance toward novelty, and mice would normally prefer dark enclosures over brightly lit open spaces. Disinhibition and anxiety have been studied before in the Tau58/2 line employing the EPM, as have behavioral paradigms with a light/dark transition chamber, fear conditioning, and cliff avoidance behavior [26,34,36], though contradictory results were reported. Whereas an anxiolytic-like phenotype was reported in Tau58/2 females and males of various ages [25,26,34], with an even more pronounced phenotype in 6- to 7-month-old female versus male Tau58/2 mice [26], another study failed to show these changes in 14-month-old females [36]. Differences in equipment and test paradigms or even different housing conditions can be responsible for site-specific differences in anxiety-related phenotypes of rodent models [66,67], but our results clearly support the development of a disinhibition/anxiolytic-like phenotype in the Tau58 lines. The EPM is most commonly considered to assess anxiety-like behavior [67,68], and ill-considered interpretation of anxiolytic effects as disinhibition-like behavior should be avoided. Disinhibition is indeed a core symptom in bvFTD, and impulsivity, compulsivity, as well as social disinhibition are documented in the diagnostic criteria of the disease [69], as such reflecting the multifaceted nature of inhibitory deficits in patients. Moreover, even only recently, it has been argued that characterization of behavioral disinhibition in patients is still poorly detailed, and a universally accepted conception is still lacking [70,71]. As such, assessing behavioral disinhibition in rodent models should include a variety of setups and paradigms mimicking the umbrella concept in patients. As previously discussed, Tau58 lines display stereotyped behavior, i.e., obsessive grooming indicative of increased compulsivity. Social disinhibition may manifest, for example, as aggressive behavior (hostility, verbal or physical aggression), inappropriate familiar or sexual behavior, as well as the loss of decorum in the clinical ethogram [70,71], which would prompt a more in-depth evaluation of various social behaviors in both lines in the future. Deficits in social exploration were reported in the Tau58/2 line from the age of 4 months onwards [26,35]. Assessments of aggressive behavior so far have been inconclusive, as only a single age group of young Tau58/2 mice was studied [35]. Impulsivity, often considered as a tendency to act prematurely without forethought or appropriate consideration of risk, also would require specific behavioral approaches in rodent models. So far, no significant differences between WT and Tau/58 mice have been reported, although also in this case, the analysis was limited to a single group of young Tau58/2 males using the cliff avoidance test [35].

We did not observe differences in traveled distance in the EPM, which could be considered as a measure of hyperactivity; however, striking differences between both Tau58 models were observed in the OFT. HET animals of the 58/4 line spent more time in the center circle and less time in the corners of the OFT area at older ages (12 months). Furthermore, despite showing significant motor impairment on the rotarod and during gait analysis, 12-month-old HET Tau58/4 mice displayed increased locomotor activity in the OFT compared to WT age-matched littermates. For the Tau58/2 model, significant differences in the OFT parameters were only found at the age of 3 months. However, HET animals also spent more time in the center circle of the OFT. Previous studies indicated that in the Tau58/2 model, HET mice showed marked differences that started at the age of 2 months and increased with age [26]. We did not find similar results suggesting that Tau58/4 mice spent the same amount of time in the center area of the OFT as WT control animals. The time spent in the center was previously attributed to be a factor for disinhibition [25] rather than an indication of decreased anxiety; however, as stated before, these statements should be interpreted with due caution.

Overall, our EPM and OFT results indicate that HET mice of both Tau58 models display altered exploration, increased activity, and less anxiety-related behavior in these paradigms. However, in a less-stressful novel environment, such as the small mouse cage applied in the 47 h cage activity recording, HET mice of both models did not exhibit hyperactivity, and no differences were observed between HET animals and controls during the first few hours of the recordings, which can be considered as exploration of the novel environment rather than a pure measurement of spontaneous circadian cage activity [47,72]. Analogous as in the OFT, we noted the development of hyperactivity in addition to altered circadian horizontal locomotion patterns with aging in HET mice of both Tau58 lines. Disrupted sleep and circadian patterns have been reported in FTD patients, which differ in nature from sleep architecture changes in other types of dementia, including AD [73,74], prompting a more in-depth study of sleep architecture in these transgenic tau models. Changes in EEG power and sleep architecture have been reported in, for example, a human P301L mutant tau mouse model [75].

The observed decrease in anxiety-related behavior and/or disinhibition conversely did not result in significant differences in passive avoidance learning as assessed with a step-through box. Using a step-down paradigm, no differences in passive avoidance performance were reported before in 4-month-old Tau58/2 mice [35]. Passive avoidance learning and memory combine emotional learning and contextual fear conditioning, which rely heavily on limbic processing in both the amygdala and hippocampus [76]. Drawbacks of the one-trial passive avoidance task, include different sensitivities to the shock exposures and memory retention, is influenced by factors like the mental state at the time of learning, emotional reactivity, and responses to stress hormones [76]. Strikingly, aged HET mice of both models appeared to display more anxiety-related behavior in the highly stressful environment of the illuminated chamber during the test trial, as evidenced by a higher number of fecal boli, pilo-erection, and trembling, which was not observed in WT control animals.

We established the age-dependent development of tau pathology in the hippocampuses of both Tau58 lines; hyperphosphorylated tau species and NFTs in the hippocampus were previously reported in the Tau58/2 line [24,26,31]. As such, we further investigated hippocampal-dependent long-term visuo-spatial learning and memory with the MWM, a commonly applied sensitive paradigm to investigate hippocampal deficits in rodents [77]. A few studies have assessed hippocampus-dependent learning and memory in the Tau58/2 line, in particular the cheeseboard test (a food-baited dry land maze) indicating an impairment in long-term spatial learning memory and defective spatial reference memory in 14-month-old females [36] and a Y-maze test indicating spatial working memory deficits in 10- to 11-month-old Tau58/2 mice, notwithstanding intact memory consolidation and retrieval [26]. One other study applied an MWM paradigm; 2-, 4-, and 6-month-old male Tau58/2 and WT mice were evaluated in a training phase with five trial blocks immediately followed by a probe trial on day 6. The onset of progressive spatial learning deficits was paralleled by long-term potentiation deficits and neuronal network aberrations. With aging, Tau58/2 mice displayed significantly increased escape latencies as well as delayed and incomplete conversion from non-spatial to spatial escape strategies. However, no genotype differences were reported in probe trial performance, indicative of intact memory consolidation and retrieval [31]. A different test paradigm (eight acquisition trial blocks and a probe trial performed only 4 days after the final training trial) and a larger-sized maze (150 cm vs. 120 cm diameter) could be used. MWM performance can be affected by various technical and non-technical variables, including apparatus characteristics, training procedure, and paradigms [77]. Our MWM assessment revealed significant differences predominantly in the oldest age group of 12 months. In both Tau58 models, HET mice showed an impaired learning curve during the acquisition phase of the MWM in that they covered longer path lengths to reach the platform, indicating that hippocampus-dependent learning was significantly impaired in both models. Path length values are the least affected by sensorimotor bias and the strongest indicators of spatial learning decline [77]. Results from the probe trial further indicated that the number of crossings over the previous platform location was significantly lower in aged HET mice of both lines in comparison with age-matched WT controls. Most importantly, swimming velocity did not differ significantly between HET and WT control animals of both Tau58 models, confirming that the observed deficits can be fully attributed to cognitive deficits and not motor bias. Our results clearly support the age-dependent development of MWM learning and retrieval in parallel with progression of hippocampal tau pathology.

## 5. Conclusions

To conclude, we established an age-dependent development of tau pathology accompanied by motor dysfunction and behavioral and cognitive impairments in both the Tau58/2 and Tau58/4 line in comparison to their WT counterparts. Both models reflect the human clinical spectrum reported in (bv)FTD, and especially FTDP-17. Our results further support the face and construct validity of both transgenic lines and their potential merit to gain further insight in tauopathies. Both models are extremely suitable to investigate both motor functions and behavior up to an older age compared to various other mouse models, thereby better mimicking the human course of the disease. Importantly, motor impairments do not interfere with important behavioral paradigms, such as the MWM, which is the case in various other tauopathy mouse models with a more aggressive motor phenotype, like for example, the widely used PS19 tauopathy model also harboring the P301S MAPT mutation. This model develops clasping and limb retraction when lifted by the tail as early as 3 months of age, followed by limb weakness progressing to paralysis at 7–10 months of age. Their median survival was reported to be only ∼9 months [78].

Thus, the Tau58/2 and 58/4 models are highly suitable to further contribute to the development of new therapeutic strategies as they aid to elucidate the link between tau pathophysiology and motor, behavioral, and cognitive decline, thereby enabling therapeutic target identification and subsequent evaluation of novel treatment possibilities.

## Figures and Tables

**Table 1:** Motor function in tau HET mice versus age-matched WT littermates as tested with the wire suspension test, stationary beam test, and gait analysis based on footprint patterns.

Tau58/2	3 Months		6 Months		9 Months		12 Months	
	WT	HET	WT	HET	WT	HET	WT	HET
Wire Suspension Test								
Number of falls	7.1 ± 1.0	7.1 ± 1.1	10.4 ±1.5	8.1 ± 0.9	9.0 ± 0.9	10.7 ± 1.0	9.3 ± 1.9	14.1 ± 1.5
Stationary Beam Test								
Number of segments	67 ± 8	47 ± 8	55 ± 7	27 ± 5 **	32 ± 6	16 ± 5	39 ± 5	16 ± 3 **
Number of falls	0.7 ± 0.4	0.8 ± 0.2	0.3 ± 0.2	1.1 ± 0.3 *	0.8 ± 0.3	2.1 ± 0.4 *	0.6 ± 0.2	2.1 ± 0.4 **
Latency to first fall (s)	205 ± 22	204 ± 12	222 ± 11	194 ± 12	206 ± 14	153 ± 19 *	213 ± 10	161 ± 18
Gait Analysis								
Stride length left (mm)	61 ± 2	53 ± 2 **	68 ± 1	56 ± 1 ***	65 ± 2	52 ± 3 ***	68 ± 4	50 ± 1 **
Stride length right (mm)	61 ± 1	52 ± 2 **	67 ± 2	58 ± 1 ***	65 ± 2	54 ± 3 **	71 ± 4	49 ± 2 ***
Toespan left (mm)	8.48 ± 0.14	8.65 ± 0.15	8.38 ± 0.16	7.79 ± 0.16 *	8.2 ± 0.15	8.16 ± 0.09	9.05 ± 0.1	8.57 ± 0.11 *
Toespan right (mm)	8.22 ± 0.13	8.39 ± 0.18	7.94 ± 0.15	7.44 ± 0.13 *	7.69 ± 0.2	7.58 ± 0.19	7.62 ± 0.22	8.04 ± 0.15
Width (mm)	27 ± 0	25 ± 1 *	27 ± 1	28 ± 0.5 *	29 ± 1	28 ± 0.5	29 ± 1	28 ± 1
**Tau58/4**	**3 Months**		**6 Months**		**9 Months**		**12 Months**	
	**WT**	**HET**	**WT**	**HET**	**WT**	**HET**	**WT**	**HET**
Wire Suspension Test								
Number of falls	7.4 ± 1.2	7.8 ± 0.8	11.6 ± 1.2	7.7 ± 1.5	9.7 ± 2.9	8.8 ± 1.7	7.8 ± 1.6	9.6 ± 2.1
Stationary Beam Test								
Number of segments	60 ± 9	20 ± 3 **	48 ± 8	14 ± 6 **	42 ± 10	10 ± 5 *	25 ± 6	6 ± 2 *
Number of falls	0.2 ± 0.1	1.5 ± 0.5 *	0.5 ± 0.3	0.9 ± 0.3	0	1.8 ± 0.4 **	0.5 ± 0.2	2.3 ± 0.3 ***
Latency to first fall (s)	232 ± 6	211 ± 10	217 ± 14	201 ± 11	233 ± 7	169 ± 19 **	222 ± 7	141 ± 15 ***
Gait Analysis								
Stride length left (mm)	63 ± 3	62 ± 2	62 ± 2	49 ± 2 ***	69 ± 2	50 ± 2 ***	72 ± 2	47 ± 1 ***
Stride length right (mm)	65 ± 3	60 ± 2	62 ± 2	50 ± 2 ***	69 ± 2	49 ± 1 ***	74 ± 1	46 ± 1 ***
Toespan left (mm)	8.51 ± 0.18	8.44 ± 0.19	8.48 ± 0.24	8.26 ± 0.17	7.64 ± 0.2	7.91 ± 0.16	8.13 ± 0.12	8.26 ± 0.14
Toespan right (mm)	8.2 ± 0.22	8.14 ± 0.25	8.77 ± 0.15	7.9 ± 0.17 **	7.89 ± 0.11	7.73 ± 0.19	7.3 ± 0.25	7.9 ± 0.17
Width (mm)	26 ± 1	26 ± 0.4	29 ± 1	28 ± 0.4	29 ± 0.5	28 ± 1	29 ± 0.4	27 ± 1 *

Data are presented as mean ± SEM. Statistical significance was determined with the two-tailed Student’s *t*-test. * = *p* < 0.05, ** = *p* < 0.01, *** = *p* < 0.001.

**Table 2:** Exploration, anxiety, and passive avoidance learning in the Tau58 mouse models.

Tau58/2	3 Months		6 Months		9 Months		12 Months	
	WT	HET	WT	HET	WT	HET	WT	HET
Elevated Plus Maze								
Time in open arms (s)	21 ± 8	117 ± 26 **	23 ± 4	100 ± 15 ***	29 ± 12	69 ± 16	15 ± 8	98 ± 20 **
Time in closed arms (s)	251 ± 12	153 ± 27 **	224 ± 8	140 ± 13 ***	240 ± 15	187 ± 15 **	259 ± 11	158 ± 18 ***
Distance (cm)	1098 ± 39	939 ± 52 *	1035 ± 59	996 ± 44	1027 ± 71	964 ± 56	1080 ± 79	925 ± 53
Open Field Test								
Time in center (s)	14 ± 4	31 ± 7 *	19 ± 3	22 ± 3	14 ± 4	27 ± 5	19 ± 5	31 ± 5
Time in corners (s)	192 ± 23	160 ± 19	185 ± 13	146 ± 10 *	220 ± 32	159 ± 10	141 ± 19	120 ± 13
Distance (cm)	2677 ± 165	3318 ± 151 *	3122 ± 162	3631 ± 154 *	2894 ± 334	3266 ± 203	3300 ± 277	3152 ± 189
Passive Avoidance Test								
Latency (s)	242 ± 35	219 ± 41	223 ± 34	262 ± 21	174 ± 26	227 ± 26	156 ± 50	226 ± 29
**Tau58/4**	**3 Months**		**6 Months**		**9 Months**		**12 Months**	
	**WT**	**HET**	**WT**	**HET**	**WT**	**HET**	**WT**	**HET**
Elevated Plus Maze								
Time in open arms (s)	8 ± 2	44 ± 13 *	46 ± 20	85 ± 26	40 ± 26	126 ± 27 *	19 ± 4	127 ± 17 ***
Time in closed arms (s)	264 ± 5	199 ± 18 **	229 ± 18	184 ± 24	230 ± 24	139 ± 25 *	242 ± 9	134 ± 16 ***
Distance (cm)	995 ± 58	956 ± 58	1274 ± 85	1214 ± 92	913 ± 77	831 ± 98	1085 ± 66	832 ± 47 **
Open Field Test								
Time in center (s)	18 ± 2	27 ± 4	12 ± 3	18 ± 2	30 ± 7	65 ± 14 *	13 ± 2	34 ± 5 ***
Time in corners (s)	199 ± 10	193 ± 12	187 ± 20	199 ± 17	153 ± 21	91 ± 15 *	193 ± 12	154 ± 10 *
Distance (cm)	3761 ± 277	4318 ± 288	3150 ± 145	3389 ± 205	3193 ± 179	3443 ± 182	3219 ± 131	3948 ± 191 **
Passive Avoidance Test								
Latency (s)	237 ± 35	288 ± 12	226 ± 29	291 ± 9	174 ± 39	198 ± 37	220 ± 31	249 ± 21

Data are presented as mean ± SEM. Statistical significance was determined with the two-tailed Student’s *t*-test. * = *p* < 0.05, ** = *p* < 0.01, *** = *p* < 0.001.

## Data Availability

The raw data that support the findings of this study are available from the corresponding author upon reasonable request.

## Appendix A

**Table A1:** Number of animals used for histological and behavioral evaluation.

Model	Tau58/2								Tau58/4							
Age	3M		6M		9M		12M		3M		6M		9M		12M	
Genotype	WT	HET	WT	HET	WT	HET	WT	HET	WT	HET	WT	HET	WT	HET	WT	HET
Histology and EM	3	3	3	3	3	3	3	3	3	3	3	3	3	3	3	3
SHIRPA, neuromotor tests, EPM, OFT and PA	10	10	15	21	13	12	7	15	9	10	11	11	11	12	15	16
MWM	9	10	13	11	10	10	10	10	13	11	10	13	10	10	12	11
Activity recordings	8	8	8	8	8	8	10	8	8	8	8	8	8	8	8	8

WT = wild-type; HET = heterozygous; EM = electron microscopy; SHIRPA = SmithKline Beecham, Harwell, Imperial College, Royal London Hospital, phenotype assessment; EPM = elevated plus maze; OFT = open field test; PA = passive avoidance test; MWM = Morris water maze test.

**Table A2:** Overview of histopathology stains conducted in both mouse models.

Line	Age	Genotype	Staining					
			AT8	AT100	AT270	Gallyas	HT7	SMI
58/2	3	WT	-	NA	-	NA	-	NA
		HET	(+)	NA	+(+)	NA	+++	NA
	6	WT	-	NA	-	-	-	-
		HET	++	NA	++	-	++	++
	9	WT	-	NA	-	NA	-	NA
		HET	++	NA	++	NA	++	NA
	12	WT	-	-	-	NA	+/-	-
		HET	+++	+++	++	NA	++	++
	15	WT	-	-	-	NA	+/-	-
		HET	+++	+++	+++	NA	+++	+++
	18	WT	-	NA	-	-	+/-	-
		HET	+++	NA	+++	+++	+++	+++
58/4	3	WT	-	NA	+/-	NA	-	NA
		HET	(+)	NA	+	NA	+++	NA
	6	WT	-	NA	-	-	-	-
		HET	+(+)	NA	+	-	++	++
	9	WT	-	NA	-	NA	-	NA
		HET	++	NA	+	NA	++	NA
	12	WT	-	-	-	NA	-	-
		HET	++	++	+	NA	++	++
	15	WT	-	-	-	NA	+/-	-
		HET	+++	++(+)	++	NA	++	++
	18	WT	-	NA	-	-	+/-	-
		HET	+++	NA	+	+++	++	++

+++ pathology observed in brainstem, midbrain, and frontal cortex; ++ pathology observed in brainstem, minor frontal cortex staining; + pathology observed in brainstem; +/- some pathology observed in brainstem and spinal cord; no pathology in brain; - no pathology observed; NA = not available; WT = wild-type; HET = heterozygous; AT8 = phosphorylated tau antibody (Ser202, Thr205); AT100 = phosphorylated tau antibody (Thr212, Ser 214); AT270 = phosphorylated tau antibody (Thr181); Gallyas = antibody to argyrophilic structures, including neurofibrillary tangles; HT7 = antibody to human tau, amino acid sequence PPGQK; SMI = antibody to neurofilament.
